# The new ceRNA crosstalk between mRNAs and miRNAs in intervertebral disc degeneration

**DOI:** 10.3389/fcell.2022.1083983

**Published:** 2022-12-02

**Authors:** Xingye Li, Yan An, Qilong Wang, Xiao Han

**Affiliations:** Department of Spine Surgery, Beijing Jishuitan Hospital, Fourth Clinical College of Peking University, Beijing, China

**Keywords:** intervertebral disc degeneration, miRNAs, ceRNAs, single-cell transcriptome sequencing, mRNAs

## Abstract

Degeneration of the intervertebral disc has been linked to lower back pain. To date, pathophysiological mechanisms of intervertebral disc degeneration (IDD) remain unclear; it is meaningful to find effective diagnostic biomarkers and new therapeutic strategies for IDD. This study aimed to reveal the molecular mechanism of IDD pathogenesis from the multidimensional transcriptomics perspective. Here, we acquired IDD bulk omics datasets (GSE67567 and GSE167199) including mRNA, microRNA expression profiles, and single-cell RNA sequencing (GSE199866) from the public Gene Expression Omnibus (GEO) database. Through principal component analysis and Venn analysis, we found different expression patterns in the IDD transcription level and identified 156 common DEGs in both bulk datasets. GO and KEGG functional analyses showed these dysregulators were mostly enriched in the collagen-containing extracellular matrix, cartilage development, chondrocyte differentiation, and immune response pathways. We also constructed a potentially dysregulated competing endogenous RNA (ceRNA) network between mRNAs and miRNAs related to IDD based on microRNA target information and co-expression analysis of RNA profiles and identified 36 ceRNA axes including ZFP36/miR-155-5p/FOS, BTG2/hsa-miR-185-5p/SOCS3, and COL9A2/hsa-miR-664a-5p/IBA57. Finally, in integrating bulk and single-cell transcriptome data analyses, a total of three marker genes, *COL2A1*, *PAX1*, and *ZFP36L2*, were identified. In conclusion, the key genes and the new ceRNA crosstalk we identified in intervertebral disc degeneration may provide new targets for the treatment of IDD.

## Introduction

Lower back pain is a leading cause of disability, affecting about 80% of individuals at least once during their life, and may lead to high healthcare costs and poor quality of life (Li et al., 2019; Chen et al., 2020a; Guo et al., 2020; Wang et al., 2021a). Degeneration of the intervertebral disc has been linked to lower back pain (Tang et al., 2020; Huang et al., 2021; Zhang et al., 2021). Intervertebral discs comprise the annulus fibrosus and nucleus pulposus (NP), and the function of the disc is to retain the vertebral stability (Wang et al., 2018a; Tan et al., 2018; Jiang et al., 2020). There are many risk factors for intervertebral disc degeneration (IDD), including genetic susceptibility, aging, smoking, heavy load work, and body weight (Wang et al., 2017a; Chen et al., 2021a). To date, the pathophysiological mechanisms of IDD remain unclear, and it is difficult to find effective diagnostic biomarkers and new therapeutic strategies for IDD.

MicroRNA (miRNA) is a subtype of noncoding, endogenous transcripts with 21–25 nucleotides in length that modulates gene expression in transcription, post-transcription, and epigenetics (Li et al., 2022; Niu et al., 2022; Xiao et al., 2022; Yang et al., 2022). Dysregulated expression of miRNA has been found to participate in many diseases such as neurological diseases, cardiovascular disease, cancer, and other diseases (Lin et al., 2019; Niu et al., 2020; Zhu et al., 2020; Andreucci et al., 2022). Recent studies have demonstrated that miRNAs play important roles in development of orthopedic diseases including IDD (Tan et al., 2021; Yang et al., 2021). Extensive studies indicated transcription RNA products such as lncRNAs and circRNAs can act as natural miRNA sponges through their miRNA response elements (MRE), acting as competing endogenous RNAs (ceRNAs) (Salmena et al., 2011). The ceRNA crosstalk plays a critical role in modulating gene expression (Thomson and Dinger, 2016). Accumulating studies have found several ceRNAs were related to autophagy, apoptosis, and cell cycle in IDD (Xi et al., 2017; Han et al., 2018; Zhan et al., 2020). Previous studies of ceRNA interactions in IDD had focused on lncRNAs and circRNAs as the sponges of miRNAs (Wang et al., 2017a; Xi et al., 2017; Cheng et al., 2018). Also, mRNAs can competitively bind to miRNAs through their MREs, thus acting as ceRNAs to regulate the expression of miRNA–target mRNAs (Tay et al., 2014; Xu et al., 2015). Nevertheless, it lacked a global view of the ceRNA crosstalk between mRNAs and miRNAs in IDD progression. It is important to evaluate the ceRNA regulation network in the type of mRNA–miRNA–mRNA crosstalk to improve our understanding of molecular mechanisms in IDD.

In this study, systematic bioinformatics analysis and expression profiles including microarrays and next-generation sequencing from bulk and single-cell transcriptome analyses were used to identify the key dysregulators in IDD. Then, combining miRNA targeting information and gene expression validation, we constructed the dysregulated ceRNA networks between miRNAs and mRNAs in intervertebral disc degeneration.

## Materials and methods

### Data acquisition

The dataset GSE67567 profiled by Arraystar Human microarray V2.0 (Agilent-033010) and GSE167199 identified by RNA sequencing (Illumina NovaSeq 6000) were downloaded from the Gene Expression Omnibus database (GEO, https://www.ncbi.nlm.nih.gov/geo/). GSE67567 and GSE167199 were composed of five and three pairs of intervertebral disc degeneration (IDD), respectively, and normal specimens as control (IDnD). For single-cell transcriptome analysis, GSE199866 was downloaded. Freshly isolated human cells were separately isolated from non-degenerating and degenerating discs of the same individual. Isolated cells were subjected to droplet-based single-cell RNA sequencing using the 10X Genomics platform. The individual cells were profiled from IDnD and IDD discs.

### Differential expression analysis

The package in R (version 4.2.1) named “limma” was used to analyze the differential gene expression (Ritchie et al., 2015). The differentially expressed genes (DEGs) between IDD and IDnD tissues were screened with the cutoff value at *p*-value < 0.05 and |Log2 FoldChange|>1. The differentially expressed mRNAs in both bulk RNA expression datasets were utilized for subsequent analysis. Meanwhile, the differentially expressed miRNAs between IDD and control tissues were also calculated as the aforementioned method.

### CeRNA construction between mRNAs and miRNAs in intervertebral disc degeneration

The miRNAs targeting mRNAs pairs were predicted using miRTarbase (Huang et al., 2022) and starBase databases (Li et al., 2014). In order to study the possible cell specific functions of the ceRNA network, we obtained the human miRNA–DEG relationships and selected some eligible pairs with gene expression validation. It requires the following conditions (Xu et al., 2015): 1). The mRNAs that shared miRNA have the positive correlation of expression (*p* < 0.05). 2).The hypergeometric distribution of the number of shared miRNAs is significant (*p* < 0.05). 3). The miRNA and target mRNA have the negative correlation of expression (*p* < 0.05).

### Protein–protein interaction network and gene functional analysis

The DEGs were mapped to the STRING database (https://cn.string-db.org/, version 11.5) to construct their PPI network (Szklarczyk et al., 2022; von Mering et al., 2003). The network visualization and analysis was conducted by Cytoscape software (https://cytoscape.org/, version 3.9.1) (Shannon et al., 2003). Gene Ontology (GO) and Kyoto Encyclopedia of Genes and Genomes (KEGG) enrichment analyses were used to analyze the biological pathways (Kanehisa and Goto, 2000). The R package named “clusterProfiler” was applied to acquire the functional enrichment terms of the DEGs (Wu et al., 2021). The annotation clusters with the *p*-value < 0.05 were treated as statistically significant. All analyses were performed by R software (version 4.2.1). The *p*-value less than 0.05 was considered statistically significant.

### Single-cell transcriptome analysis

The single-cell datasets were integrated using Seurat’s alignment procedure (Hao et al., 2021). Canonical correlation analysis (CCA) was performed to identify shared sources of variation to produce anchors across the datasets, following SCTransform normalization and principal component analysis for feature extraction. An unsupervised nonlinear dimensionality reduction technique was based on the first 30 principal components. Visualization was performed *via* Uniform Manifold Approximation and Projection (UMAP).

## Results

### The different transcriptome expression pattern in IDD

**FIGURE 1:**
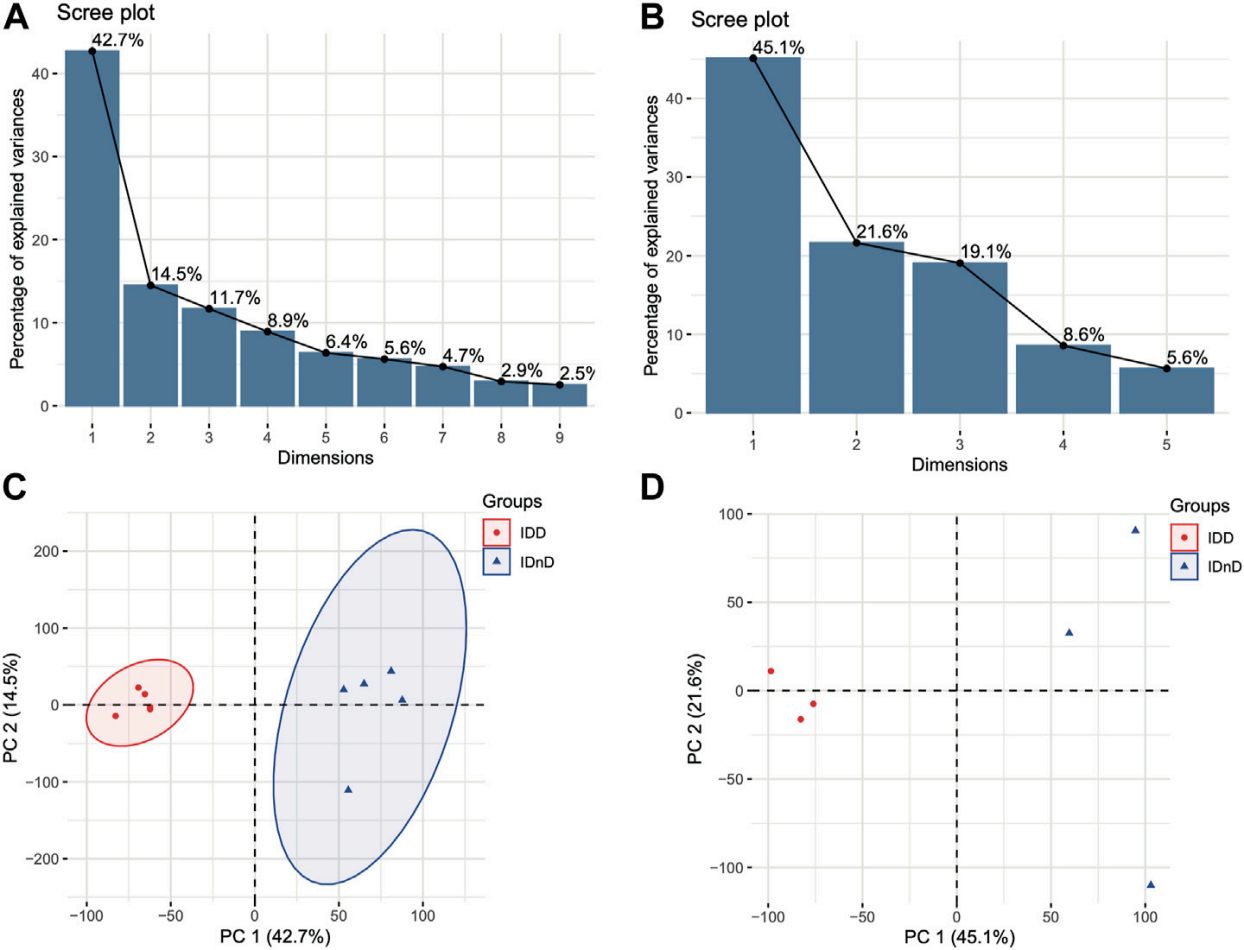
Different transcriptome expression patterns between IDD and IDnD. **(A)** Scree plot of the principal components in the GSE67567 dataset. **(B)** Scree plot of the principal components in the GSE167199 dataset. **(C)** PCA and clustering analysis in the GSE67567 dataset. **(D)** PCA and clustering analysis in the GSE167199 dataset. Axes are principal components 1 and 2.

**FIGURE 2:**
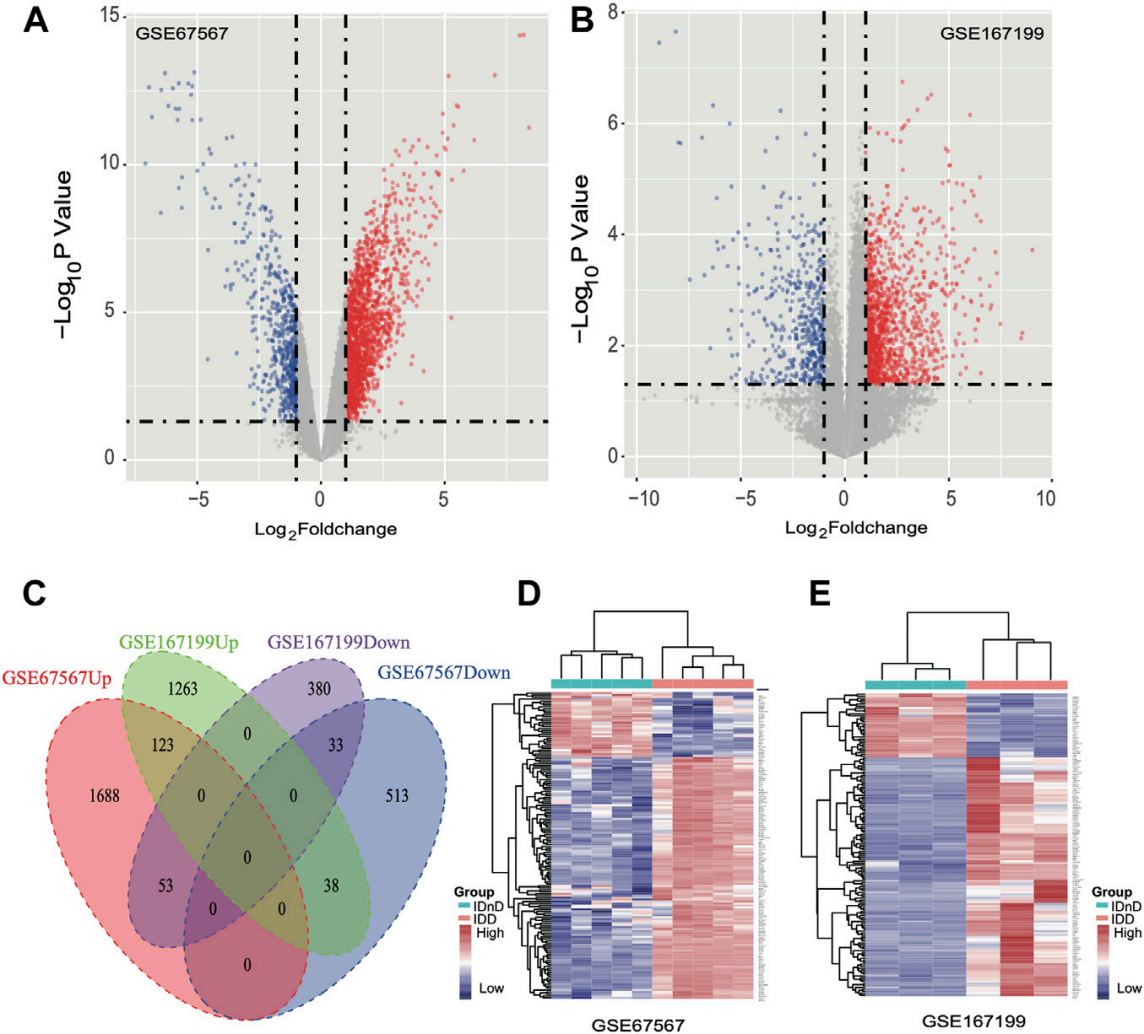
Differentially expressed mRNAs between IDD and IDnD. **(A)** Comparison of differential mRNA volcano plots between nucleus pulposus samples from IDD and IDnD in the GSE67567 dataset. **(B)** Comparison of DEG volcano plots between nucleus pulposus samples from IDD and IDnD in the GSE167199 dataset. **(C)** Venn diagram of the DEGs from both datasets. **(D)** Expression heatmap of 156 common DEGs in the GSE67567 dataset. **(E)** Expression heatmap of 156 common DEGs in the GSE167199 dataset.

Based on the mRNA expression profiles from bulk datasets GSE67567 and GSE167199, we identified transcription patterns of intervertebral disc degeneration (IDD) samples and the normal controls (intervertebral disc, non-degeneration, and IDnD), respectively. The result of the aforementioned datasets indicated that IDD was different from IDnD in the transcription level using principal component analysis (PCA) (Figures 1A,B) and clustering (Figures 1C,D). Then, the differentially expressed gene analysis (*p* < 0.05, |Log2 FoldChange|>1) of microarray data GSE67567 showed that a total of 2448 mRNAs were significantly differentially expressed in IDD compared with IDnD. There were 1,864 upregulated mRNAs and 584 downregulated mRNAs (Figure 2A, Supplementary Figure S1A). As for the RNA-seq dataset GSE167199 (Figure 2B, Supplementary Figure S1B), it contained 1890 DEGs (1424 downregulated and 466 upregulated). By Venn analysis, 156 common DEGs were in the same expression trend in both datasets (33 downregulated and 123 upregulated, Figure 2C). The expression heat maps of the common 156 DEGs are visualized in Figure 2D (GSE67567) and Figure 2E (GSE167199). In the same way, we identified a total of six significantly differentially expressed miRNAs including hsa-miR-3131, hsa-miR-3150a-3p, hsa-miR-4731-3p, hsa-miR-4741, hsa-miR-486-5p, and hsa-miR-642b-3p (Table 1).

**TABLE 1:** Significantly differentially expressed miRNAs in both datasets.

MicroRNA name	GSE67567		GSE167199	
	logFC	*p*-value	logFC	*p*-value
hsa-miR-3131	1.36	0.04	4.44	0.00
hsa-miR-3150a-3p	3.07	0.02	4.96	0.00
hsa-miR-4731-3p	1.59	0.02	1.78	0.01
hsa-miR-4741	1.99	0.00	4.61	0.02
hsa-miR-486-5p	−1.60	0.01	−1.60	0.02
hsa-miR-642b-3p	1.37	0.00	5.00	0.02

### Functional analysis of the DEGs

**FIGURE 3:**
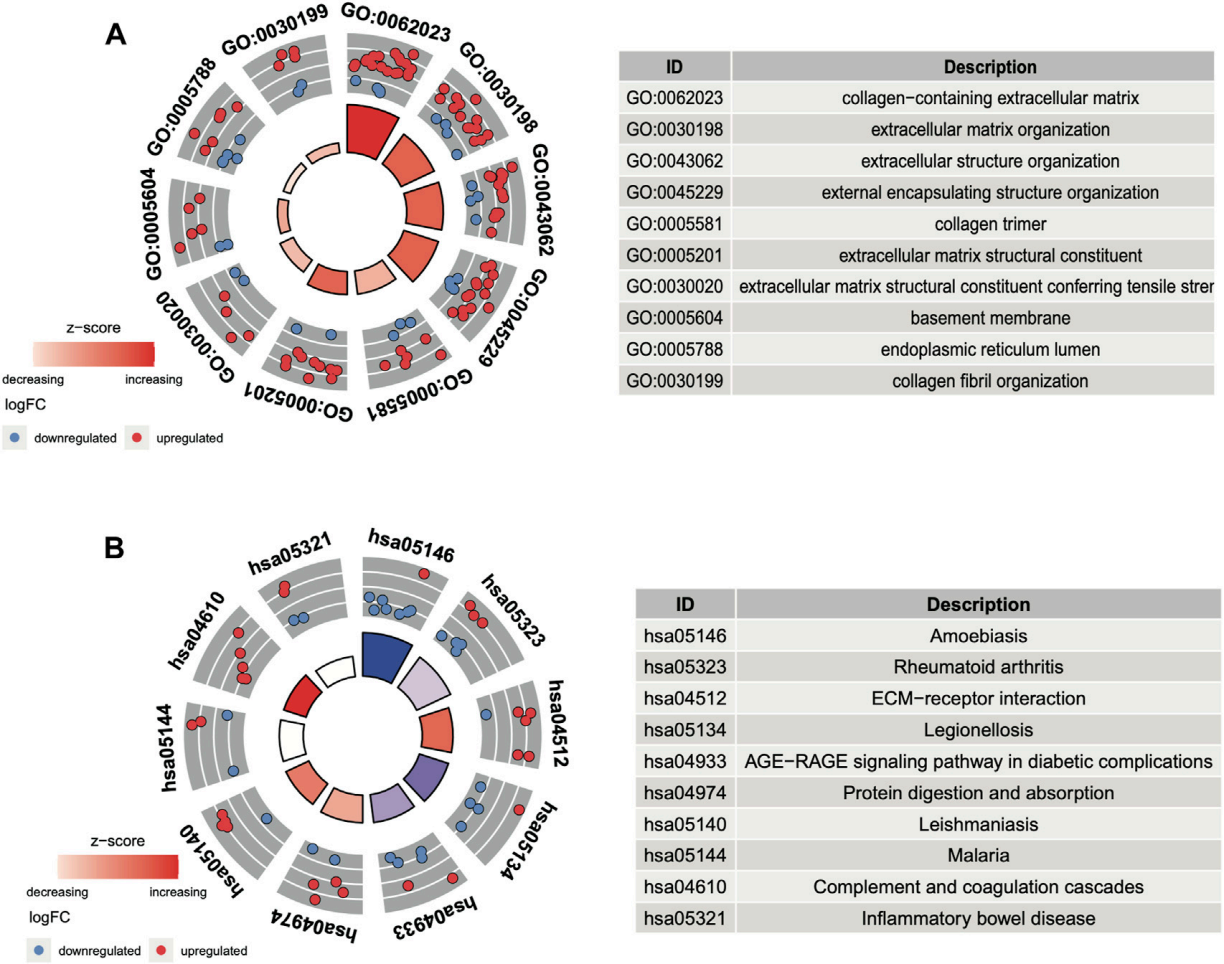
Functional analysis of DEGs. **(A)** Top 10 GO terms ranked by the *p*-value. **(B)** Top 10 KEGG pathways ranked by the *p*-value.

In GO and KEGG analyses by the R software package clusterProfiler, we identified 73 enriched GO terms and 41 KEGG pathways from the aforementioned 156 common DEGs (*p* < 0.05, Supplementary Table S1). The top 10 GO terms ranked by the *p*-value are shown in Figure 3A. The most enriched GO term in BP was the “GO:0030198-extracellular matrix organization” (*p* < 0:001, *n* = 18); in CC was the “GO:0062023-collagen-containing extracellular matrix” (*p* < 0:001, *n* = 23); and in MF was the “GO:0005201-extracellular matrix structural constituent” (*p* < 0:001, *n* = 12). There are more upregulated genes than downregulated genes, suggesting that the GO terms may be activated. The top 10 enriched KEGG pathways of the DEGs are presented in Figure 3B. The most enriched pathways were associated with amoebiasis, rheumatoid arthritis, and the ECM–receptor interaction.

### Construction of the PPI network of the DEGs

**FIGURE 4:**
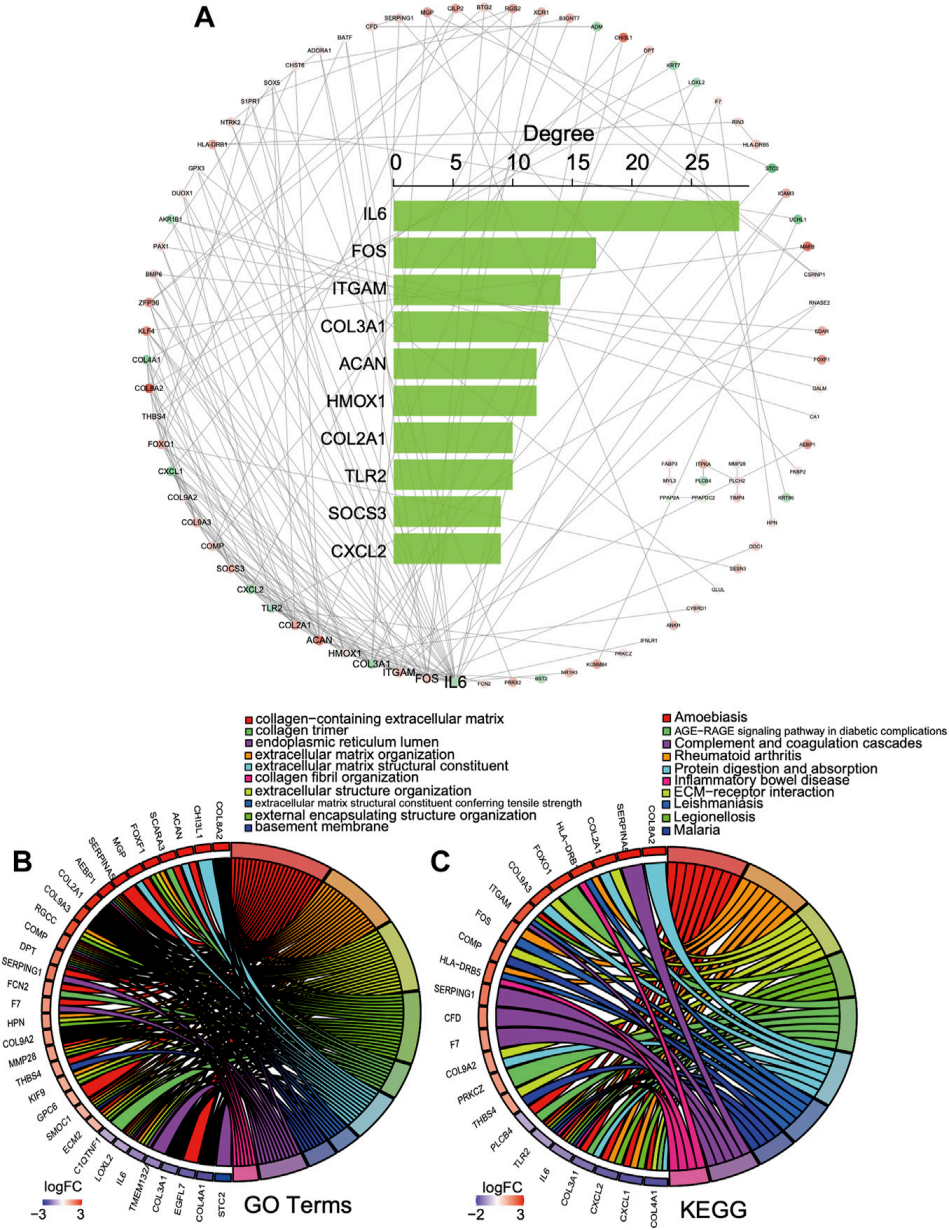
PPI network of the DEGs. **(A)** PPI network and top 10 gene degree distribution: red represents upregulated, and blue represents downregulated. **(B, C)** Hub gene relations in the top 10 GO terms and KEGG pathways.

According to the common 156 DEGs with the same expression trend in both bulk datasets, a PPI network was established including 83 nodes and 167 interacting pairs (Supplementary Table S2). After degree analysis by Cytoscape, the top 10 genes in terms of degree named *IL6*, *FOS*, *ITGAM*, *COL3A1*, *ACAN*, *HMOX1*, *COL2A1*, *TLR2*, *SOCS3*, and *CXCL2* were considered hub genes in the network (Figure 4A). It has been reported that they were all involved in progression intervertebral disc degeneration (Osuka et al., 2014; Lv et al., 2016; Johnson et al., 2017; Makino et al., 2017; Lin et al., 2018; Chen et al., 2019; Wu et al., 2019; He et al., 2021; Li et al., 2021). As shown in Figure 4, the hub genes in the top 10 GO terms (Figure 4B) and KEGG pathway (Figure 4C) were closely associated with the collagen-containing extracellular matrix, ECM−receptor interaction, and immune regulation.

### Construction of the dysregulated competing endogenous RNA between mRNAs and miRNAs

**FIGURE 5:**
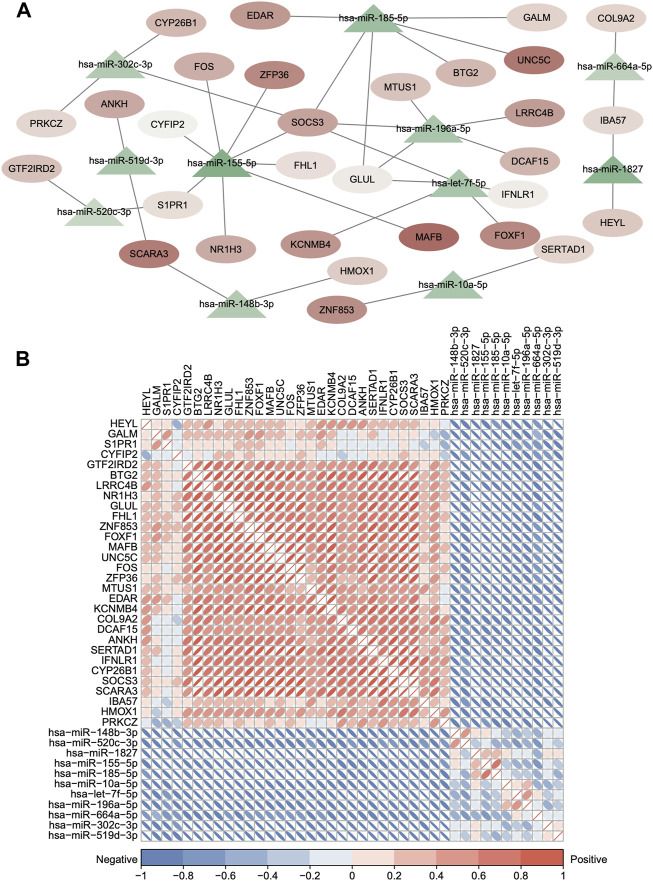
Dysregulated ceRNA between mRNAs and miRNAs in IDD. **(A)** Dysregulated ceRNA network between miRNAs and differentially expressed mRNAs. (hypergeometric test *p* < 0.05 and Pearson test *p* < 0.05). Red represents upregulated, and blue represents downregulated. The triangle nodes represent DEGs, and ellipse nodes represent miRNAs; the color gradient indicates the difference in expression. **(B)** Clustered corrplot of miRNAs and differentially expressed mRNAs in the ceRNA network. The color gradient represents the expression correlation score between genes.

First, we obtained 5,705 human miRNA–DEG relationships from miRTarbase and starBase based on the 156 common DEGs. Second, the hypergeometric test was used to evaluate the number of shared miRNAs among the DEGs with *p* < 0.05, and the dysregulated ceRNA network was constructed which contained 308 miRNAs, 133 DEGs, and 1728 interacting pairs (Supplementary Figure S2A). Finally, the expression correlation score of genes was calculated by the Pearson test, and we set the *p*-value < 0.05, and 36 ceRNA axes were screened (Figure 5A, Supplementary Table S3). We used these qualified relationships to construct the ceRNA network and visualize it by Cytoscape software. This ceRNA network includes 39 edges with 30 DEGs and 12 miRNA nodes. The triangles nodes represent DEGs, ellipse nodes represent miRNAs, and the color gradient indicates the difference in expression (Figure 5A). The DEGs that shared same miRNAs show strong positive correlation of expression in Figure 5B in red color. At the same time, some DEGs are clustered in the specific set, proving that they may have synergistic functions, such as *ZFP36*, *SOCS3*, and *FOS*. On the contrary, the miRNA and target DEGs show strong negative correlation of expression in Figure 5B in blue color. All the aforementioned results can prove the reliability of the ceRNA network and the regulatory function of the miRNA in the target gene directly or indirectly.

### Integrating single-cell and bulk transcriptome analyses

**FIGURE 6:**
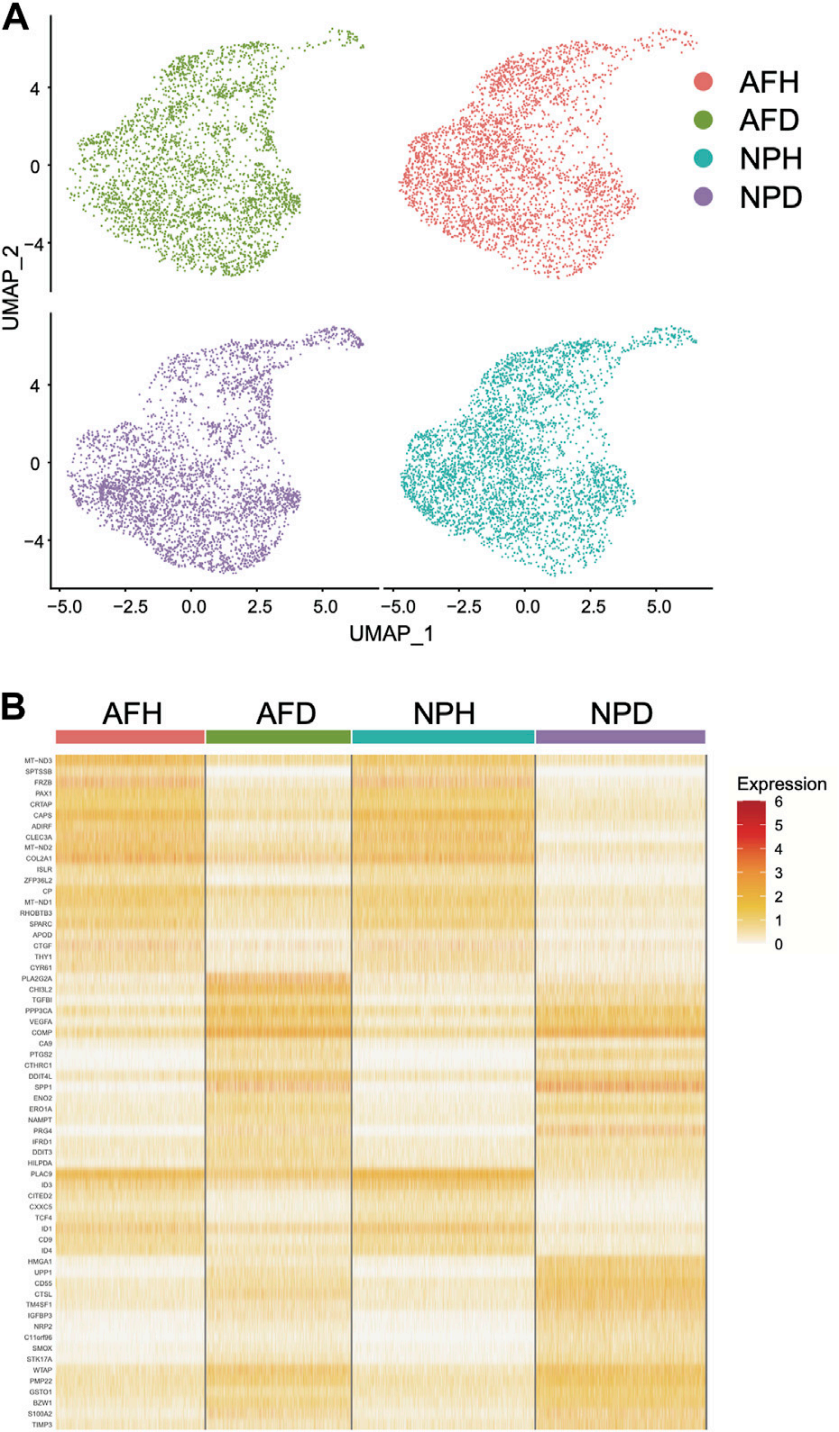
Single-cell transcriptome analysis. **(A)** Single-cell clustering in the annulus fibrosus (AF) and nucleus pulposus (NP) of patients with intervertebral disc degeneration (IDD). **(B)** Heatmap of marker gene expression in each cell group (top 10).

**FIGURE 7:**
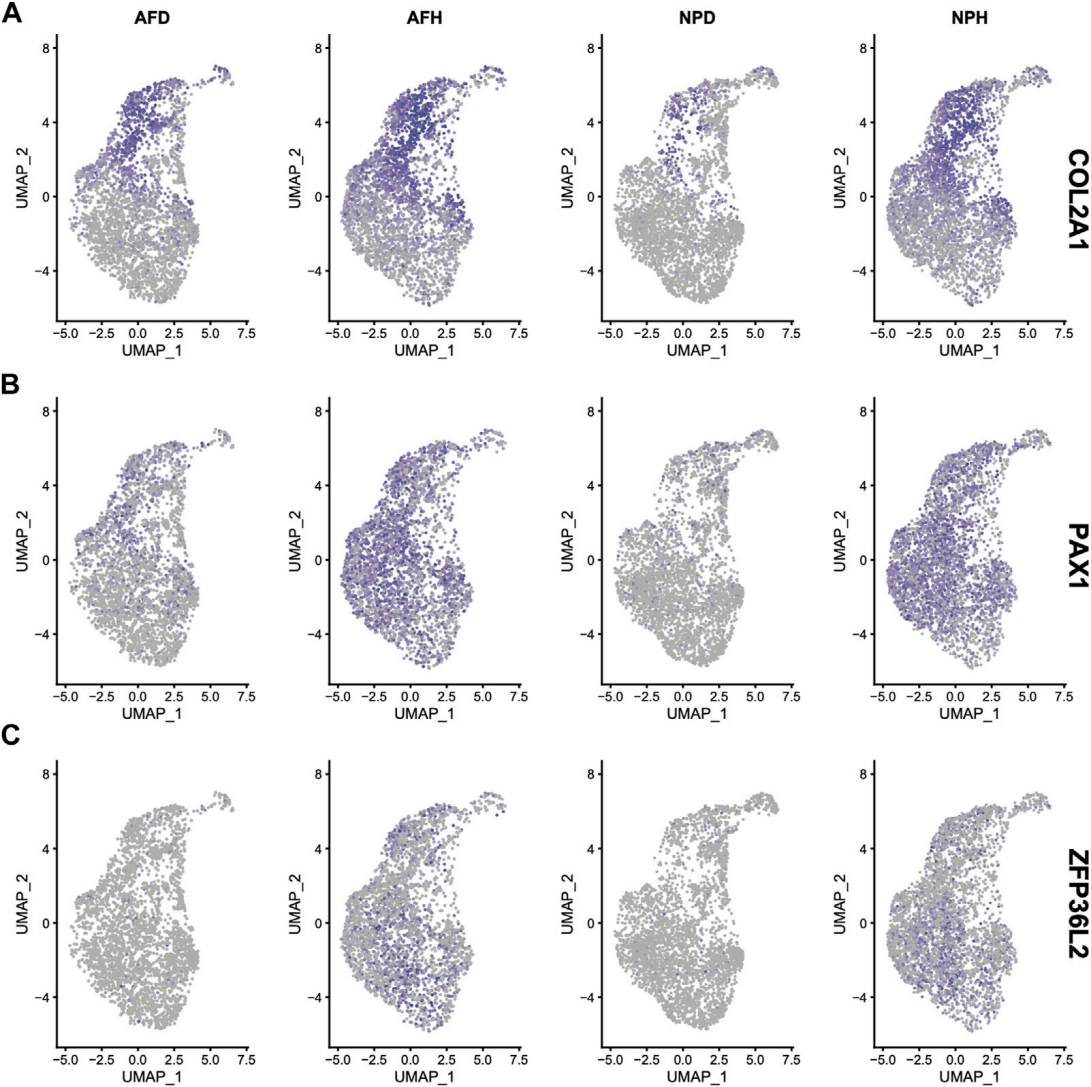
Expression of *COL2A1* **(A)**, *PAX1* **(B)**, and *ZFP36L2* **(C)** in the four single-cell samples.

Single-cell clustering in the annulus fibrosus (AF) and NP of patients with IDD is shown in Figure 6A. We obtained 115 marker genes (AF = 30, AFH = 18, NPH = 26, and NPD = 41, Supplementary Table S4) based on 3,142 AF cells from degenerated discs (AFD), 3,226 AF cells from non-degenerated discs (AFH), 3,678 NP cells from degenerated discs (NPD), and 3,955 NP cells from non-degenerated discs (NPH). A heatmap of the top 10 marker gene expressions in each cell group is shown in Figure 6B. Intersection analysis of the marker genes was conducted with the aforementioned 156 bulk DEGs and showed three common differential genes including *COL2A1* (Figure 7A), *PAX1* (Figure 7B), and *ZFP36L2* (Figure 7C).

## Discussion

Intervertebral disc degeneration (IDD) is an age-related chronic degeneration with NP cell senescence and imbalance between extracellular matrix (ECM) catabolism and synthesis (Shao et al., 2019; Chen et al., 2021a; Chen et al., 2021b). With the disc degeneration, the synthesis of proteoglycan and collagen I and degradation of the ECM are increased (Chen et al., 2020b; Cui et al., 2020; Gao et al., 2020). Although IDD is an age-induced condition, it is also influenced by other risk factors including genetic susceptibility, aging, smoking, heavy load work, and body weight (Wang et al., 2017b; Wang et al., 2018b; Xiao et al., 2020). Several biotechnologies were shown to abate IDD by inducing ECM regeneration and repair (Wang et al., 2018c; Yang et al., 2020). Thus, it is important to find effective diagnostic biomarkers and new therapeutic strategies for IDD.

Recently, several studies have demonstrated that miRNAs play important roles in many biological processes such as cell apoptosis, ECM degradation, and cell proliferation (Wang et al., 2021b; Yu et al., 2021; Zhou et al., 2021). For example, Zhang et al. (2022) demonstrated that miR-4478 induced apoptosis of NP cells through targeting MTH1. Cui et al. (2022) showed that miR-760 prevented IDD development by targeting the NF-κB signaling pathway and MyD88. Zhou et al. (2022) showed that miR-206 ameliorated IDD through targeting GJA1. Yu et al. (2022) demonstrated that miR-137 suppressed ECM degradation and inflammatory response in lipopolysaccharide-induced NP cells through targeting ACVR1. Chen et al. (2022a) indicated that miR-1260b ameliorated LPS-induced IDD development by targeting TCF7L2. Cao et al. (2021) demonstrated that miR-200c-3p inhibited IDD development through targeting the RAP2C/ERK signaling pathway. Guo et al. (2021) demonstrated that miR-502 inhibited TNF-α-induced NP cell apoptosis through targeting TARF2. These results suggested that miRNAs play important roles in the development of IDD. We found six significantly differentially expressed miRNAs in both datasets, hsa-miR-3131, hsa-miR-3150a-3p, hsa-miR-4731-3p, hsa-miR-4741, hsa-miR-486-5p, and hsa-miR-642b-3p. The role of these miRNAs in IDD has not yet been proven in other studies.

Through Venn analysis, we found 156 DEGs had same expression trends in both bulk datasets. Further GO and KEGG functional analyses showed these dysregulators were mostly enriched in the collagen-containing extracellular matrix, cartilage development, chondrocyte differentiation, and immune response pathways. Moreover, it showed that several dysregulated genes played important roles in the PPI network, including genes that were related to cytokines (*IL6*, *SOCS3*, *CXCL2*, *CXCL1*, and *TLR2*), complements (*ITGAM*), ECM composition including the collagen family, and aggrecan (*COL3A1*, *COL2A1*, *COL9A2*, *COL9A3*, *COL4A1*, *COL8A2*, *ACAN*, and *COMP*), as well as cell proliferation or differentiation transcription factors (*FOS* and *FOXO1*). In previous studies, collagen and aggrecan turnover in ECM was observed in degenerated discs (Sivan et al., 2014; Trefilova et al., 2021). COMP, the cartilage oligomeric matrix protein, was also found as a marker of IVDD (Qi et al., 2021). Several cytokines including IL1, IL5, IL6, IL7, IL10 TLR4, and TNF-alpha were found to promote extracellular matrix degradation, leading to the degeneration of IVD (Klawitter et al., 2014; Osuka et al., 2014; Risbud and Shapiro, 2014). Macrophage infiltration was also observed in herniated discs, which may cause a variable level of *ITGAM* expression (Kawaguchi et al., 2001). FoxO1a was found to mediate apoptosis in disc degeneration (Jing et al., 2021). FOS was proved to regulate inflammatory response in IVDD (Makino et al., 2017).

The GO and KEGG analyses in our study also demonstrated multiple pathways related to ECM metabolism and immune response. However, KEGG analysis also demonstrated pathways involving parasites (amoebiasis, malaria, and leishmaniasis) or bacterial infection (legionellosis). The reason was that IVDD and parasite or certain bacterial infection have overlap in pathways that involves cytokine release (Cruz Cubas et al., 1994; Friedman et al., 1998; García-Zepeda et al., 2007; Samant et al., 2021) and ECM degradation (Meza, 2000).

The mRNA–miRNA–mRNA pattern of the ceRNA network has been found in cancers (Tay et al., 2014; Xu et al., 2015); however, it has not been observed in IDD yet. We constructed a potentially dysregulated ceRNA network between mRNAs and miRNAs related to IDD based on microRNA target information and co-expression analysis of RNA profiles and identified 36 new ceRNA axes (Supplementary Table S3 and Figure 5A). Some genes involved in the axes were proved functional in IDD, including ZFP36/miR-155-5p/FOS, BTG2/hsa-miR-185-5p/SOCS3, and COL9A2/hsa-miR-664a-5p/IBA57 (Osuka et al., 2014; Meng et al., 2016; Makino et al., 2017; Xu et al., 2022) by regulating ECM metabolism and immune response; however, the function of more axes still remains unclear.

Single-cell transcriptome sequencing is a new method to delineate heterogeneous tissues at the single-cell level (Chen et al., 2022b; Galuzzi et al., 2022; Muhl et al., 2022). By integrating bulk and single-cell transcriptome data analyses, a total of three marker genes, *COL2A1*, *PAX1*, and *ZFP36L2*, were identified. Pax1 was found to be an important transcription factor in the development of the intervertebral disc (Sivakamasundari et al., 2017; Nakamichi and Asahara, 2020). However, no study was found describing how ZFP36 relates to disc degeneration. ZFP36 was differentially expressed and was a hub gene in GO and KEGG analyses. Its ligand, ZFP36L2, was found differentially expressed in single-cell transcriptome analysis. Zinc finger protein 36 (ZFP36) family proteins are RNA-binding proteins involved in mRNA metabolism pathways (Makita et al., 2021). It plays a significant role in regulating immune responses and inflammatory diseases (Lai et al., 1999) and cancers (Schuschel et al., 2020). This is a pathway worthy of further investigation.

In summary, the key genes and the new ceRNA crosstalk we identified in intervertebral disc degeneration may provide new understanding of the molecular mechanism of IDD pathogenesis from the ceRNA perspective and may provide new targets for treating IDD.

## Data Availability

The datasets presented in this study can be found in online repositories. The names of the repository/repositories and accession number(s) can be found in the article/Supplementary Material.
