# Supplementary figures and images for: The new ceRNA crosstalk between mRNAs and miRNAs in intervertebral disc degeneration
Source: Front Cell Dev Biol. 2022 Dec 2;10:1083983. doi: 10.3389/fcell.2022.1083983 (PMC9755594; doi:10.3389/fcell.2022.1083983)

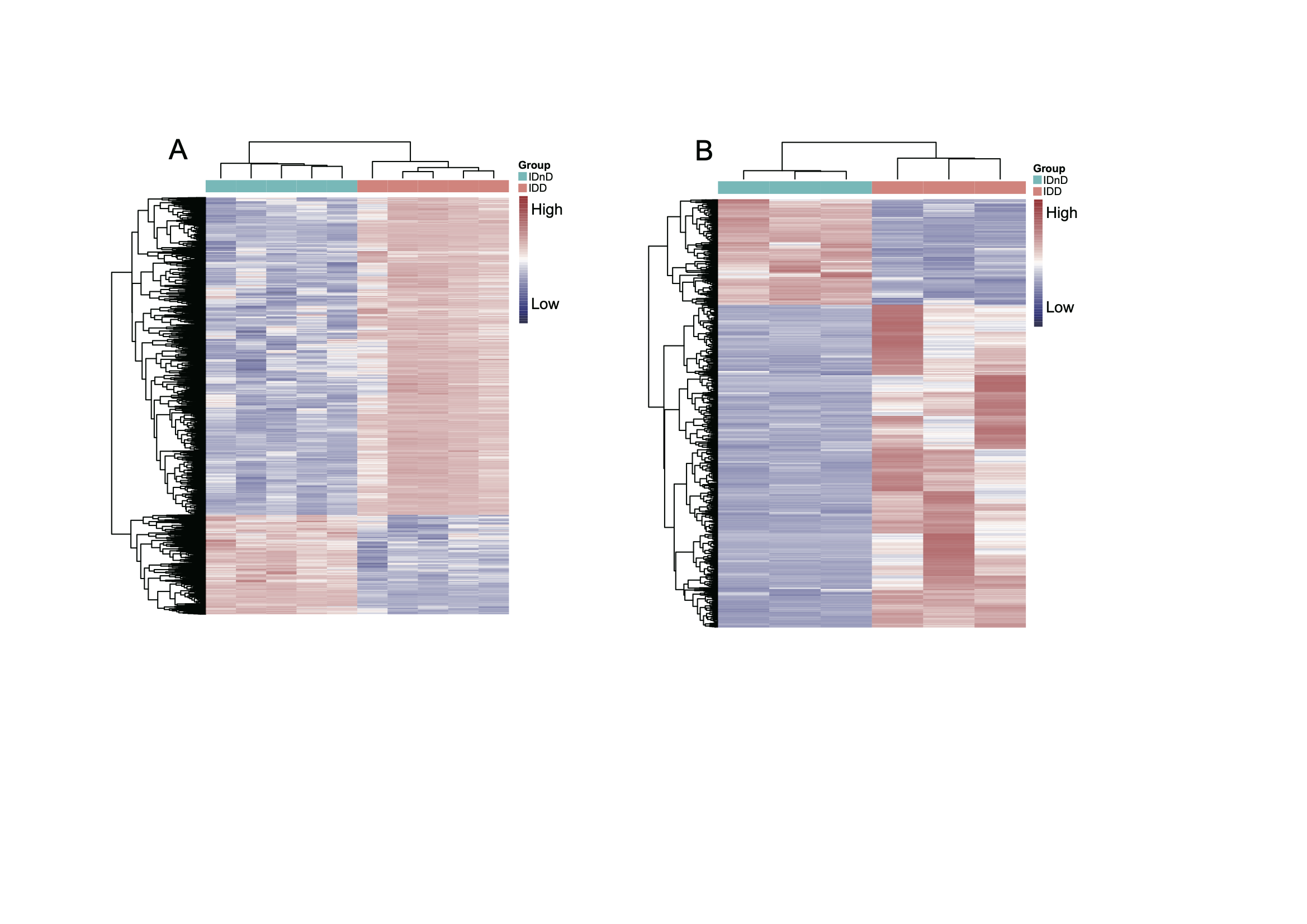

Supplement: Supplementary file 2 [file Image1.TIFF]

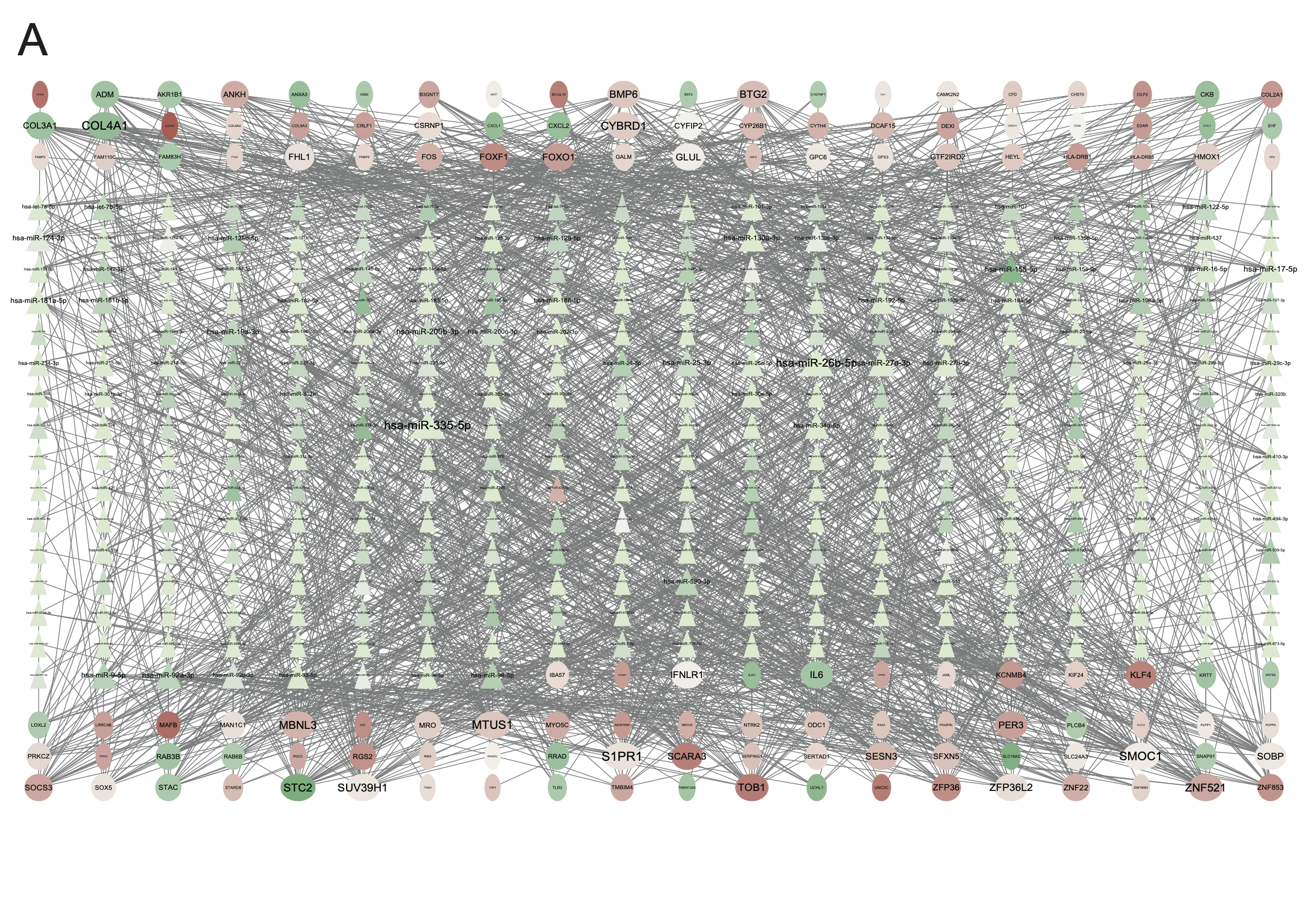

Supplement: Supplementary file 3 [file Image2.TIFF]
